# Valuing the Unpaid Contribution of Community Health Volunteers to Mass Drug Administration Programs

**DOI:** 10.1093/cid/ciy741

**Published:** 2018-08-29

**Authors:** Hugo C Turner, Jaspreet Toor, Alison A Bettis, Adrian D Hopkins, Shwe Sin Kyaw, Obinna Onwujekwe, Guy E Thwaites, Yoel Lubell, Christopher Fitzpatrick

**Affiliations:** 1 Oxford University Clinical Research Unit, Wellcome Trust Major Overseas Programme, Ho Chi Minh City, Vietnam; 2 Centre for Tropical Medicine and Global Health, Nuffield Department of Medicine, University of Oxford; 3 London Centre for Neglected Tropical Disease Research, School of Public Health, Faculty of Medicine, St Marys Campus, Imperial College London; 4 Department of Infectious Disease Epidemiology, School of Public Health, Faculty of Medicine, St Marys Campus, Imperial College London; 5 Independent Consultant, Gravesend, Kent, United Kingdom; 6 Mahidol Oxford Tropical Medicine Research Unit, Faculty of Tropical Medicine, Mahidol University, Bangkok, Thailand; 7 Department of Health Administration and Management, University of Nigeria Enugu Campus; 8 Health Policy Research Group, Department of Pharmacology and Therapeutics, College of Medicine, University of Nigeria Enugu Campus, Enugu, Nigeria; 9 Department of Control of Neglected Tropical Diseases, World Health Organization, Geneva, Switzerland

**Keywords:** community volunteers, economic costs, unpaid work, mass drug administration, APOC

## Abstract

Community health volunteers (CHVs) are being used within a growing number of healthcare interventions, and they have become a cornerstone for the delivery of mass drug administration within many neglected tropical disease control programs. However, a greater understanding of the methods used to value the unpaid time CHVs contribute to healthcare programs is needed. We outline the two main approaches used to value CHVs’ unpaid time (the opportunity cost and the replacement cost approaches). We found that for mass drug administration programs the estimates of the economic costs relating to the CHVs’ unpaid time can be significant, with the averages of the different studies varying between US$0.05 and $0.16 per treatment. We estimated that the time donated by CHVs’ to the African Programme for Onchocerciasis Control alone would be valued between US$60 and $90 million. There is a need for greater transparency and consistency in the methods used to value CHVs’ unpaid time.

## Teaser

The global health community needs a greater understanding of the opportunity costs community health volunteers incur when contributing to healthcare programs. Focusing on mass drug administration programs as an example, we found that these costs can be significant.

Mass drug administration (MDA) is used to control several of the most prevalent neglected tropical diseases (NTDs). Originally, the treatments were distributed by mobile teams of paid, local health professionals [1]. However, due to the costs of this, control programs shifted to using trained volunteers from the community [1, 2]. The African Programme for Onchocerciasis Control (APOC) pioneered a community-directed MDA strategy in which communities themselves direct the planning and implementation of the delivery of the treatments [2–4]. By 2014, the APOC had a network of over 699656 volunteer community-directed distributors [4]. The strategy has become widely recognized as instrumental to the tremendous progress achieved in the control and elimination of onchocerciasis [3].

Volunteer community drug distributors are now used to deliver treatments to hundreds of millions of people each year for onchocerciasis and lymphatic filariasis [5], and they are also being used to support MDA for malaria control [6]. Recently there has been increased interest in using this approach to broaden the control of schistosomiasis and soil-transmitted helminths from schools into communities [7]. Besides MDA, using community health volunteers (CHVs; Box 1) have been identified as a potential solution to curbing human resource shortages in healthcare in rural areas [8], and they are being used within a number of other healthcare interventions: such as vitamin A supplementation, supporting patients with human immunodeficiency virus (HIV) and tuberculosis, and community case management of childhood illnesses [3, 9–14]). In addition, many Ministries of Health are becoming increasingly committed to using community health workers (such as in Rwanda, where they have developed a large national community healthcare worker program [15]). In some countries the concept of CHVs as “volunteers” is becoming blurred, as they have become increasingly integrated within health systems and are being offered more formal compensation.

**Box 1. Glossary**

**Agriculture value added per worker metric:** Agriculture value added per worker is a measure of agricultural productivity, estimated by the World Bank [16]. Agriculture comprises value added from forestry, hunting, and fishing as well as cultivation of crops and livestock production.

**African Programme for Onchocerciasis Control (APOC):** The APOC was initiated in 1995 in 19 African countries to expand and build on the successes of the Onchocerciasis Control Programme in West Africa (OCP) [17]. In 1997, it adopted community-directed treatment with ivermectin as its core strategy in which a trained volunteer from the community distributes the drugs [17]. The APOC gradually expanded and when it stopped at the end of 2015, it was supporting onchocerciasis control and elimination activities in 31 African countries (including the 19 original signatories of the Memorandum, South Sudan, and 11 ex-OCP countries) [17].

**Community health workers (CHWs):** The umbrella term “community health worker” (CHW) embraces a variety of types of community health aides working in their local community [18]. In some settings, they are given a salary or stipend, whereas in others they are volunteers (what we are referring to as community health volunteers) [19].

**Community health volunteers (CHVs):** CHVs provide basic health services in their local communities. They are increasingly recognized as an integral component of the health workforce in low/middle-income countries, and as a way to help address healthcare worker shortages in these settings. Also referred to as unpaid community health workers (CHWs), community drug distributors, and community-directed distributors. Though CHVs are not given a salary, in many settings they are given external monetary incentives to motivate effort and help improve their performance. These can take the form of cash payments (such as travel and lunch allowances, stipend, or per diem) or in-kind incentives (such as bicycles) [20].

**Economic costs (opportunity costs):** Economic costs define the cost of a resource in terms of its value in its next best alternative use (also known as an opportunity cost). This is a broader conceptualization of a resource’s value than its financial cost, as it recognizes that using a resource makes it unavailable for productive use elsewhere. When the market price accurately represents a resource’s value, it can generally be assumed to reflect its economic/opportunity cost (in which case the resource’s financial and economic cost are the same). However, when this is not the case (such as for nonmarketed/donated resources) a different estimated value of the resource is used to reflect its economic/opportunity cost (often referred to as a “shadow price”). The rationale behind economic costs is that they represent the full value of all the resources used for an intervention, and they account for the fact that resources can have a value that is not fully captured by their financial costs. This is particularly important when considering issues related to the sustainability and replicability of interventions.

**Financial costs**: The actual expenditure on the goods/services purchased.

**Indirect costs (productivity costs):** Indirect costs represent the value of the productivity losses that result from illness, treatment, or premature death.

**Market price/wage:** The current prevailing price at which a good or service can be bought or sold as determined by supply and demand.

**Mass drug administration (MDA):** Where treatment is given at a large scale to eligible populations within an endemic area, without diagnosing or testing the individual participants for the infection.

**Nominal cost:** Values have not been adjusted for inflation.

**Shadow price/wage:** The estimated value of a good or service for which no market price exists.

**The opportunity cost approach:** Measures the value of a volunteer’s unpaid time in terms of the value of the next best alternative activity they have forgone in order to volunteer (such as losing the opportunity to work).

**The replacement cost approach:** Measures the value of the volunteer’s unpaid time based on what it would cost to hire a paid worker to perform the same tasks. Also known as the ‘proxy good method’ and the ‘substitute method’.

When performing economic evaluations of healthcare interventions, it is standard practice to use what are known as “economic costs” [21]. These economic costs (described further in Box 1) represent the full value of all resources used for an intervention, including the value of donated resources. These are important when considering issues related to the sustainability and replicability of interventions, which is particularly relevant for unpaid CHVs, as relying on them for such a growing range of roles and interventions could become unsustainable. Consequently, the value of the unpaid time that CHVs donate to healthcare interventions needs to be estimated and included as an economic cost within relevant economic evaluations. However, the economic costs relating to CHVs’ unpaid time are often overlooked or estimated inconsistently [22, 23]. This variation in methodology was also observed within a literature review on the costs and cost-effectiveness of community health workers programs [14].

Due to the need to intensify NTD interventions and the increasingly significant role that CHVs have within global health, it is important to understand the methods used to place an economic value on the unpaid time that CHVs contribute to healthcare interventions. Within this paper we discuss this issue, focusing on MDA for the NTDs as a case study.

## THE AMOUNT OF UNPAID TIME CHVS CONTRIBUTE TO MDA PROGRAMS

Below we summarize 3 of the main methods used to quantify the amount of unpaid time that CHVs contribute to MDA programs. These approaches are not mutually exclusive, and a combination of the methods can be used [24].

- Retrospective questionnaires: The amount of time that CHVs spend on MDA program activities can be estimated via retrospective questionnaires. A limitation of this approach is that because it is retrospective, the estimates are subject to recall and response bias.
- Direct observations: The time that CHVs spend on certain NTD program activities can be directly observed and recorded. An advantage of this method is that the information is recorded in real time, which minimizes recall bias. However, a potential limitation is that the estimates would be subject to participant bias, that is, CHVs may change their typical behavior because they are being observed. Additionally, the time CHVs spend on activities that cannot be easily observed (such as the time spent on community sensitization) would need to be estimated using other methods.
- Diaries/timesheets: This method (using pictorial diaries) was recently used to estimate the time CHVs’ spend on MDA program activities [24]. A key advantage of this method is that it reduces recall bias, though a potential limitation is that it is more labor intensive to implement, potentially resulting in smaller sample sizes.

**Figure 1.**
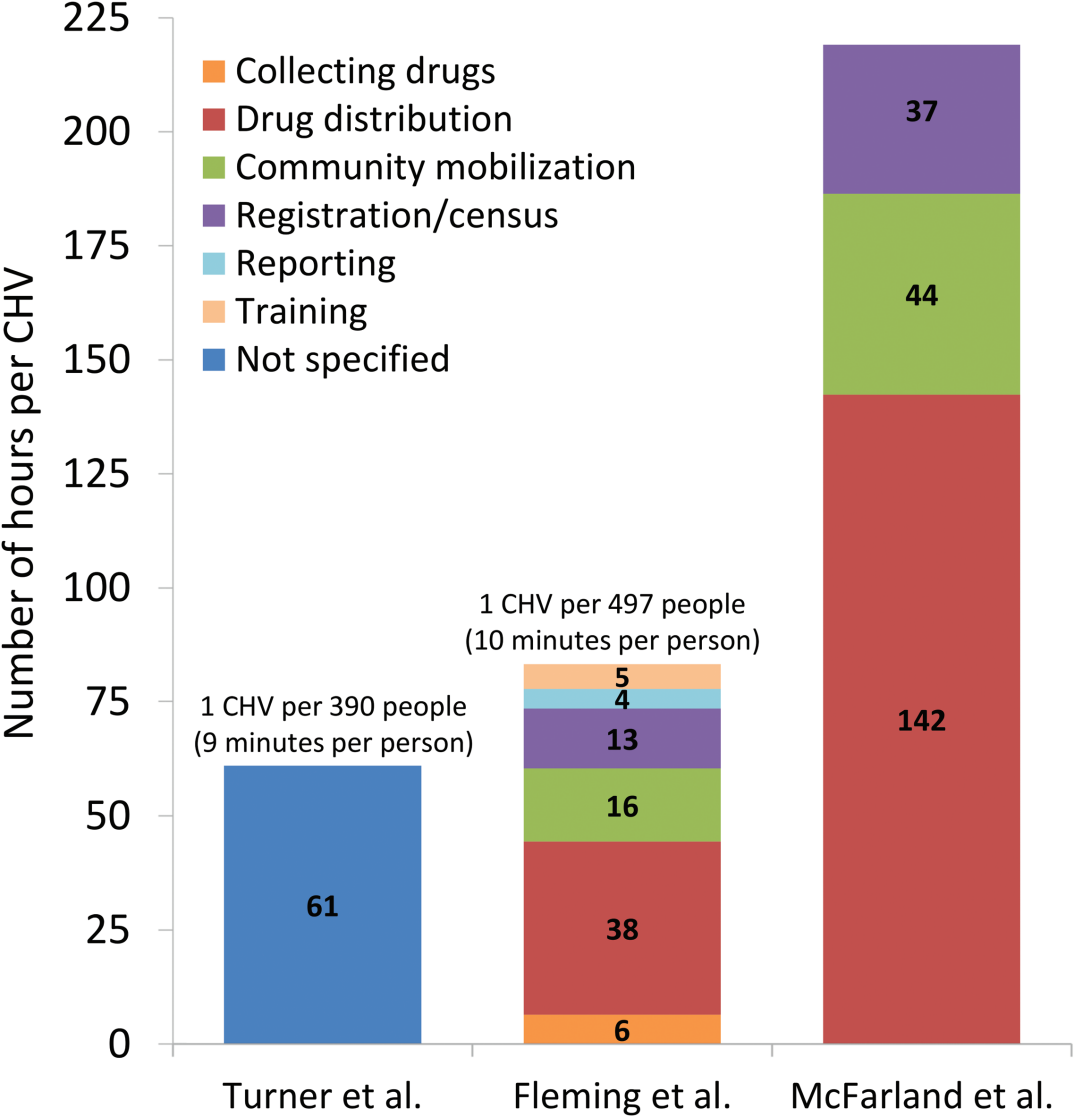
The average number of hours a CHV spends on MDA program activities. Values presented pertain to one annual delivery round of MDA. The data are adapted from the following studies: Turner et al. [25], Fleming et al. [24], and McFarland et al. [26]. Abbreviations: CMV, community health volunteer; MDA, mass drug administration.

Several of the reported estimates regarding the amount of time that CHVs spend on MDA program activities are shown in Figure 1. The values ranged between 61 and 223 hours per annual delivery round of MDA. Importantly, the amount of time that CHVs spend on the actual distribution of the drugs is only one of the activities they perform for MDA programs (Figure 1). For example, the time spent on reporting and community mobilization was also significant (Figure 1). This is important because, if this is not recognized, studies may not quantify the time spent on these other activities, underestimating the total time that CHVs donate to MDA programs.

The potential factors that may influence the amount of time that CHVs spend on MDA program activities are summarized in Box 2.

**Box 2. Potential Factors That May Influence the Amount of Time CHVs’ Spend on MDA Program Activities**

- **Number of individuals covered per CHV**: The number of individuals that a CHV covers has been found to be a significant determinant of the total time they spend on the MDA programs [24] (although it does not necessarily affect the average amount of time spent per person covered/treated) (Figure 1). This has important implications regarding how these estimates should be generalized to other settings. The APOC recommended a standard of 1 volunteer community-directed distributor per 100 people [27]. However, in practice, the population covered per volunteer varied significantly and was typically higher than this recommendation [2, 28–30] (Figure 1). Katabarwa et al. [30, 31] found that the performance of the CHVs was enhanced when each CHV was given a smaller population to cover within existing traditional kinship structures.
- **Population density**: In settings that have a low population density, it may take CHVs longer to cover the same number of people, particularly when using a house-to-house dispersal method.
- **Division of different tasks**: The division of different tasks/roles amongst the CHVs within a community will also affect the total time required for an MDA round.
- **Cultural factors**: Local cultural factors may influence the time required to distribute the drugs.
- **Community sensitization**: The level of community sensitization that has occurred could influence the amount of time it takes CHVs to distribute the drugs.
- **The targeted NTDs**: MDA is used to control various NTDs. It is possible that the amount of time that CHVs need to spend on MDA will vary depending on which NTDs are being targeted and which drugs they are distributing. In addition, Fleming et al. [24] found that the amount of time that CHVs spent on NTD control depended on the number of deliveries that were required within the integrated preventive chemotherapy campaign. In some programs, the different MDA rounds may not be scheduled at the same time of the year, likely increasing the time commitment required from the CHVs.
- **Screening**: If any screening or testing is performed prior to treatment it will likely increase the amount of time it takes CHVs to distribute the drugs.
- **The program’s phase**: A CHV may need to spend longer on MDA related activities during the start of a control program. It is also conceivable that during the final phase of the program the time that CHVs need to spend may increase due to program fatigue and declining interest from the community in receiving the treatments.
- **The support they receive:** The time a CHV needs to spend on MDA related activities will likely be influenced by the amount of support they receive from other members of the community (such as village leaders) and from the ministry of health/organization leading the intervention.

Interestingly, a study in Uganda found that the amount of time that CHVs spent on MDA program activities was not statistically related to the distribution method (door to door versus from a focal point), any sociodemographic variables (such as the CHVs’ sex, age, and marital status), their education level, occupation or length of tenure as a CHV [24], though it is unclear how generalizable this is to other settings.

## METHODS TO VALUE CHVS’ UNPAID TIME

In the following section, we outline the key methods that can be used to place an economic value on CHVs’ unpaid time. It is worth noting that though there is significant overlap with the principles and methodology used for valuing volunteers’ time, as with patients and informal caregivers indirect costs (productivity costs; Box 1) [32–34], they are not necessarily identical.

### The Opportunity Cost Approach

The opportunity cost approach is the most common method for valuing the unpaid time that CHVs contribute to MDA programs. This method measures the value of a volunteer’s unpaid time in terms of the value of the next best alternative activity they have forgone in order to volunteer (such as losing the opportunity to work) [10, 32, 34, 35]. It therefore measures the economic value of the volunteers’ time from their own perspective [34, 36].

**Figure 2.**
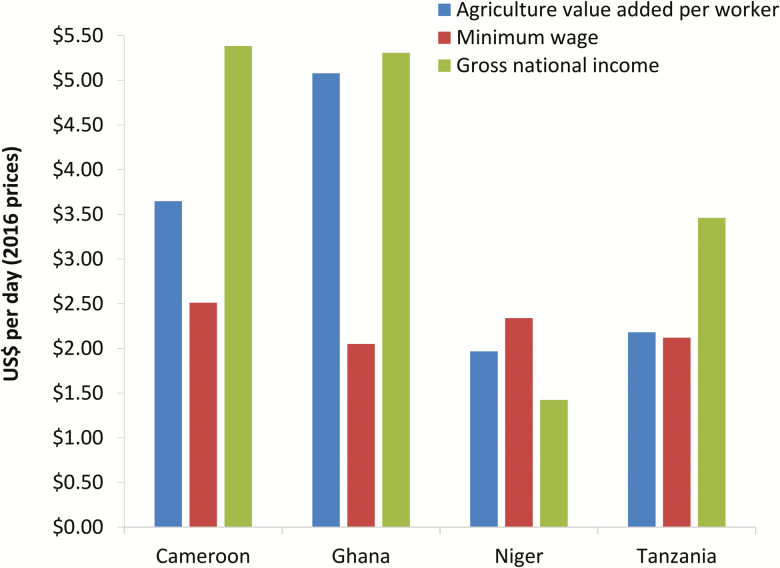
Potential daily shadow wage rates for CHVs. The values were adjusted to daily rates by assuming 260 work days per year. The values are expressed in 2016 US$ prices and were adjusted for inflation using the country’s GDP deflator [41] (accounting for changes in the US$ exchange rate [42]). The data was taken from: Agriculture value added per worker [16], minimum wage [43], and gross national income [40]. Abbreviations: CMV, community health volunteer; GDP, gross domestic product.

Because many CHVs are not in formal employment (many are subsistence farmers), estimating the opportunity cost of their time can be challenging [10, 35, 37, 38]. The simplest and most common method for using the opportunity cost approach to value CHV’s time is to apply a shadow wage rate that corresponds to the average potential earnings of the volunteering population [10, 34]. The average wage of a farmland worker/laborer is often used, as this is the most commonly reported occupation of CHVs in many settings [10, 24, 25, 39]. In some settings, this will be the same as or at least similar to the minimum wage [25] (Figure 2). An alternative shadow wage rate for farmland workers that has been used is the “agriculture value added per worker” metric, estimated by the World Bank [16, 28] (Box 1). In contrast, some studies have used the per capita gross national income (GNI) [40] as the shadow wage rate for CHVs—which represents the average income of a country’s citizens. However, this may not be representative of the income level in rural areas (Figure 2).

It should be noted that the World Health Organization’s (WHO’s) guide to cost-effectiveness analysis [21] recommends valuing nonscarce, unskilled labor from those typically engaged in agricultural production by using the local rural wage rate (adjusted for seasonal fluctuations in demand) as a proxy for the value of lost production.

The variation across some of the different potential shadow wage rates is illustrated in Figure 2. This demonstrates that the estimated value of CHVs unpaid time can be highly sensitive to the source of the shadow wage rate (Figure 2). This variation can occur even when the different shadow wage rates for a given setting relate to the same type of profession [44]. In addition, a noteworthy aspect of the opportunity cost method is that it leads to different values for the same amount of volunteered work depending on the local CHVs potential earnings. Hence it is important that shadow wage rates and estimates obtained using the opportunity cost method are not overgeneralized.

One challenge of using the opportunity cost approach to value volunteers’ unpaid time is deciding what to consider as the main activity they are giving up in order to volunteer [34]. This can be complicated for CHVs, as their work is often seasonal. Some have argued that volunteers are typically giving up their leisure time and not the opportunity to work [10, 34]. However, how to value lost leisure time is under debate within the health economic field [10, 34, 45]. It has been argued that when calculating the productivity costs (indirect costs; Box 1) associated with a disease or an intervention, lost leisure time need not be valued [45–47]. However, it is important to consider that the rationale for including the value of CHVs’ unpaid time as an economic cost is to reflect the intervention’s true cost, indicating its sustainability and replicability. Therefore, the arguments for not valuing lost leisure time when calculating productivity costs do not necessarily apply when calculating the economic value of the volunteers’ unpaid contribution to healthcare interventions. Due to the difficulties in valuing lost leisure time, it is often implicitly assumed that the alternative activity for a CHV volunteering is work, that is, the total amount of unpaid time the CHVs contribute to the MDA program is valued by the shadow wage rate, and the calculations do not distinguish between lost paid/unpaid work and lost leisure time.

It should be highlighted that even when there is seasonality in work patterns/employment, it does not necessarily mean that individuals are just having “leisure time” in the off-period. Instead they will likely change to a different type of informal employment or conduct unpaid work.

An additional limitation of the opportunity cost approach is that it does not account for the possibility that CHVs may be willing to provide their time at below market rates (or even without payment) due to the perceived benefits, both intrinsic and extrinsic, that may result from their volunteering (discussed further in Kasteng et al. [10]). In addition, in some cases the CHVs are compensated by the community, and this is typically not accounted for. CHVs contributing to MDA programs have been found to be particularly motivated by intrinsic incentives (such as recognition, status, and knowledge gain, etc.) [3, 23]. However, motivation by extrinsic (material) incentives has also been found to be a factor [3, 23]. There can be expectations that the MDA programs will provide monetary incentives to the CHVs (which is partly due to the fact that sometimes other interventions/programs offer them) [3, 23]. There is a need for more research on the role of incentives in motivation, retention, and performance of CHVs [23].

### The Replacement Cost Approach

The replacement cost approach measures the value of the volunteer’s unpaid time based on what it would cost to hire a paid worker to perform the same tasks [10, 32, 34]. An advantage of this approach is that it values CHVs’ unpaid time based on the type of work they are providing, as opposed to their alternative employment opportunities, arguably providing a better representation of the value of CHVs’ contribution from the healthcare program’s perspective [10]. A disadvantage of this approach is that it implicitly assumes that paid workers would require the same amount of time to perform the tasks as the volunteers [32, 33], which may not always be accurate. Additionally, it does not account for the possibility that a CHV may not be able to perform the tasks to the same level that a paid worker could, which could result in future costs to the healthcare system in the long term. However, the contrary is also possible, as the CHVs could be more committed than paid workers due to their genuine motivation regarding the delivery of healthcare to their community.

It is noteworthy that the WHO’s guide to cost-effectiveness analysis recommends using the replacement cost approach for valuing volunteer labor that cannot be assumed to be available indefinitely [21]. The guide states that effectively this means that volunteer labor would often be valued at the wage rate of the healthcare workers that would normally be employed to do the same tasks [21]. Kasteng et al. [10] also considered the replacement cost approach more suitable than the opportunity cost approach for valuing CHVs’ unpaid time.

Although we agree that the replacement approach may be more suitable when it is clear what type of paid worker would normally be employed to do the same tasks, we would argue that it is difficult to implement for interventions such as MDA. This is because in most settings MDA at its current scale would not be feasible without using volunteers, making it difficult to know what type of worker would normally be employed to deliver the treatments. Interestingly, if it was assumed that local laborers rather than employed healthcare workers would be used, the replacement cost approach and opportunity cost approach would yield similar estimates, as they would both be based on the same type of shadow wage rate, that is, that of a local farmland worker/laborer (Figure 2).

### Reported Estimates Within the Literature

A summary of the key estimates regarding the average economic costs relating to CHVs’ unpaid time for MDA programs is shown in Table 1. The average estimates from the different studies varied between US$0.05 and $0.16 per treatment (nominal prices). In comparison, the financial costs of MDA delivery are typically reported to be between US$0.10 and $0.50 per treatment [22]. Comparisons to these benchmarks illustrate the importance of the economic costs relating to CHVs’ unpaid time for NTD control.

**Table 1.** The Estimated Economic Costs Relating to CHVs’ Unpaid Time for Mass Drug Administration Programs

Study	Country	Method/Approach	Assumed Shadow Wage Rate	Average Economic Cost of CHVs’ Unpaid Time (Nominal Prices)	Year of Prices
[25]	Ghana	Opportunity cost	Agricultural wage (equivalent to the minimum wage in the study setting)	US$0.046 per treatment	2011
[48]	Nigeria	Opportunity cost	Minimum wage	US$0.125 per treatment	1998
[26]	Cameroon, Nigeria, Uganda	Opportunity cost	GNI	Overall average: US$0.16 per treatment ○ Nigeria: US$0.13 per treatment ○ Uganda: US$0.16 per treatment ○ Cameroon: US$0.35 per treatment	2003
[39]	Niger	Opportunity cost	Agricultural wage	US$0.05–0.07 per treatment	2005
[24]	Uganda	Opportunity cost	Laborer wage, minimum wage, GNI.	The average cost per CHV for one delivery round: ○ Laborer wage: US$28.06 ○ Minimum wage: US$20.25 ○ GNI: US$38.58 Assuming 70% treatment coverage this would correspond to: ○ Laborer wage: US$0.08 per treatment ○ Minimum wage: US$0.06 per treatment ○ GNI: US$0.11 per treatment	2010
[3]	Cameroon, Nigeria, Uganda	Opportunity cost	Minimum wage	US$44 per community treated. Assuming an average community size of 884 people^a^ and a 70% treatment coverage, would correspond to an average of US$0.071 per treatment. The total cost per community was observed to vary substantially across the different study sites.	2005
[49]	Niger	Opportunity cost	Agricultural wage	The data pertaining to CHVs alone was not shown.	-
[50]	The Philippines	Replacement cost	The average allowance typically provided to volunteer health workers	The data pertaining to CHVs alone was not shown.	-

Abbreviations: CHV, community health volunteers; GNI, gross national income.

^*a*^ Based on Kim et al. [28]. Nominal prices: The values have not been adjusted for inflation.

The range in the reported values was in part due to differences in the shadow wage rates used (Table 1). Unsurprisingly, the highest reported value used the per capita GNI as the shadow wage rate. It should be highlighted that when using the opportunity cost approach, the appropriate shadow wage rate will depend on the local CHV population. Due to missing methodological information and differences in the way the results were presented, at times it was difficult to compare the different estimates. It should be clarified that these values only reflect the economic costs of the CHVs’ unpaid time that is contributed to MDA programs. In many cases the CHVs will also be contributing to other programs; therefore, the total value of their unpaid work within global health interventions would be higher.

In Box 3 we summarize key recommendations for future studies in this area to allow for greater consistency. Although these are focused on MDA programs, they are also relevant for other interventions.

**Box 3. Recommendations for Future Studies**

- Report the average number of people covered per CHV, and the number of CHVs per community for each study setting.
- State the achieved coverage level and the number of people treated for each study setting.
- Stratify the time that CHVs spend on an intervention by the different programmatic activities they are performing (such as collecting drugs, drug distribution, reporting, etc.).
- Clearly state if the opportunity cost or replacement cost approach is being used and the specific shadow wage rate that is being assumed (reporting the actual values) for each study setting:
o When using the opportunity cost approach, justify the decision regarding the shadow wage rate and the employment opportunities of CHVs.
o When using the replacement cost approach, clearly justify the decision regarding who is assumed to be hypothetically hired to replace the CHVs.
- Regardless of the method used, illustrate how the chosen shadow wage rate compares to the other potential sources/rates in the investigated study settings.
- Clearly state any adjustments made to the wage rate. For example, if it was converted from an annual to a daily rate, indicate how many work days per year were assumed.
- Collect the financial costs that are incurred by CHVs.
- When possible, also quantify the economic costs incurred by other members of the community.
- Clearly report the estimated economic costs that have been incurred by the CHVs (ie, do not only report them within the overall personnel cost).
- Report the CHVs’ out-of-pocket financial costs and the allowances they receive separately from the economic value of their unpaid time.
- Take care to avoid double counting costs, such as the allowance given to CHVs and the out-of-pocket financial costs/economic costs they incur.

The achieved coverage level will affect the estimated per treatment economic cost of the CHVs’ unpaid time. This is because CHVs will spend time visiting households, regardless of whether the occupants take the treatment (eg, some occupants may refuse treatment or be absent at the time of the visits). Therefore, as the communities’ adherence levels decrease, the average economic cost of CHVs’ unpaid time per treatment will increase.

A study compared the economic costs incurred by CHVs when using them within an expanded strategy of “community-directed interventions” (CDI) versus using them to only distribute ivermectin within an MDA program [3]. The median economic cost for the CHVs per community was US$65 in the CDI trial communities and US$44 in the comparison (control) communities [3], although the difference was not statistically significant. The total cost per community was observed to vary substantially across the different study sites [3].

## THE TOTAL ECONOMIC COST RELATING TO THE UNPAID TIME CHVS’ DONATED TO THE APOC

Between 1997 and 2015, the APOC helped support the delivery of over one billion treatments using CHVs [4, 5] (Box 1). If it was assumed that the average economic cost relating to the CHVs’ unpaid time was between US$0.06 and $0.09 per treatment, this would result in an estimate of the total value of the CHVs’ unpaid contribution to the APOC of between US$60 and $90 million. In comparison, Coffeng et al. [51] estimated the financial cost of the APOC (1995–2015) was approximately US$478 million (nominal cost; Box 1). Therefore, we estimate that CHVs may have contributed as much as 19% of the financial cost of the program.

Although our calculation is simplistic and does not account for integrated NTD treatment programs, or the economic costs related to the unpaid time contributed by other community members (such as village leaders), it does clearly show the significance and order of magnitude of the endemic communities’ contribution to the APOC. Further research and data are necessary for more refined estimates of the total economic value of the contribution of endemic communities to NTD control globally.

## CONCLUSIONS

CHVs are being used for a growing number of healthcare interventions, and they have become a cornerstone for the control of many NTDs. However, the global health community needs a greater understanding of the value of CHVs’ unpaid time and the economic costs that CHVs incur when contributing to healthcare programs, as this is currently being widely overlooked. Focusing on MDA as an example, we found that these costs can be significant, with the averages of the different studies varying between US$0.05 and $0.16 per treatment. We estimated that the total economic cost relating to the unpaid time CHVs contributed to the APOC would be valued between US$60 and $90 million. Our calculations highlight that the endemic communities themselves are making significant commitments and contributions to NTD control and demonstrate the importance of valuing the unpaid time donated by CHVs.

It may become unsustainable to depend on CHVs for such a growing number of interventions, particularly for NTD control programs, which when fully scaled up, will cover over a billion people. It is therefore important to include the value of their unpaid time as an economic cost within economic evaluations, as it allows the sustainability of programs to be more accurately assessed and the required activities to be appropriately measured in terms of cost.

## Notes

***Financial support*.** H. C. T. and G. E. T. are supported by the Wellcome Trust [089276/B/09/7]. J. T. was supported by the Bill and Melinda Gates Foundation in partnership with the Task Force for Global Health through the NTD Modelling Consortium [OPP1053230] and the Children’s Investment Fund Foundation (UK) (“CIFF”). The views, opinions, assumptions, or any other information set out in this article are solely those of the authors and should not be attributed to the funders or any person connected with the funders.

***Potential conflicts of interest.*** All authors: No reported conflicts of interest. All authors have submitted the ICMJE Form for Disclosure of Potential Conflicts of Interest. Conflicts that the editors consider relevant to the content of the manuscript have been disclosed.
